# Shiga Toxin (Stx) Type 1a and Stx2a Translocate through a Three-Layer Intestinal Model

**DOI:** 10.3390/toxins15030207

**Published:** 2023-03-09

**Authors:** Rebecca A. Bova, Andrew C. Lamont, Theodore J. Picou, Vincent B. Ho, Kristin H. Gilchrist, Angela R. Melton-Celsa

**Affiliations:** 1 Department of Microbiology and Immunology, Uniformed Services University, Bethesda, MD 20814, USA; 2 Center for Biotechnology (4DBio3), Department of Radiology and Radiological Sciences, Uniformed Services University, Bethesda, MD 20814, USA; 3 The Geneva Foundation, Tacoma, WA 98402, USA

**Keywords:** tissue model, translocation, Shiga toxin, *Escherichia coli*

## Abstract

Shiga toxins (Stxs) produced by ingested *E. coli* can induce hemolytic uremic syndrome after crossing the intact intestinal barrier, entering the bloodstream, and targeting endothelial cells in the kidney. The method(s) by which the toxins reach the bloodstream are not fully defined. Here, we used two polarized cell models to evaluate Stx translocation: (i) a single-layer primary colonic epithelial cell model and (ii) a three-cell-layer model with colonic epithelial cells, myofibroblasts, and colonic endothelial cells. We traced the movement of Stx types 1a and 2a across the barrier models by measuring the toxicity of apical and basolateral media on Vero cells. We found that Stx1a and Stx2a crossed both models in either direction. However, approximately 10-fold more Stx translocated in the three-layer model as compared to the single-layer model. Overall, the percentage of toxin that translocated was about 0.01% in the epithelial-cell-only model but up to 0.09% in the three-cell-layer model. In both models, approximately 3- to 4-fold more Stx2a translocated than Stx1a. Infection of the three-cell-layer model with Stx-producing *Escherichia coli* (STEC) strains showed that serotype O157:H7 STEC reduced barrier function in the model and that the damage was not dependent on the presence of the *eae* gene. Infection of the three-layer model with O26:H11 STEC strain TW08571 (Stx1a+ and Stx2a+), however, allowed translocation of modest amounts of Stx without reducing barrier function. Deletion of stx2a from TW08571 or the use of anti-Stx1 antibody prevented translocation of toxin. Our results suggest that single-cell models may underestimate the amount of Stx translocation and that the more biomimetic three-layer model is suited for Stx translocation inhibitor studies.

## 1. Introduction

Shiga-toxin (Stx)-producing *E. coli* (STEC) are typically ingested from contaminated food or water or, in limited cases, transmitted directly from an infected individual [1]. The most serious STEC infections are characterized by severe abdominal pain, bloody diarrhea, and in 5–15% of cases, hemolytic uremic syndrome (HUS) [1]. HUS consists of hemolytic anemia, thrombocytopenia, and renal failure. Both the bloody diarrhea and the renal damage are likely caused by direct effects of Stx action on the endothelial cells that line small blood vessels as well as the indirect effects of the inflammatory response [2]. STEC are not invasive, so the Stxs induce systemic disease by traveling from the intestine through the bloodstream to localize to sites such as the kidney or central nervous system where the toxin receptor is abundantly expressed. There are two immunologically distinct types of Stx produced by *E. coli*, Stx1 (same as Stx from *Shigella dysenteriae* type 1) and Stx2. STEC may produce either toxin or both toxin types. Because there are several subtypes of each toxin, the prototype toxin for each group is designated Stx1a or Stx2a [3]. The Stxs are composed of a single enzymatically active A subunit that is responsible for the cytotoxic activity and a non-covalently linked pentamer of B subunits that mediates receptor binding [4]. Stx1a and Stx2a use globotriaosylceramide (Gb3) as the preferred receptor to enter host cells [5]. Gb3 is present on endothelial cells in the kidney, central nervous system, and, in limited quantities, on human colonic epithelial cells [5,6]. The Stxs also exhibit a limited capacity to bind alternate receptors such as globotetraosylceramide, Gb4 [7] and Toll-like receptor 4 (TLR4) [8].

Although a few therapeutic approaches to inhibit Stx activity have advanced to clinical trials, no specific therapy is currently available to treat STEC-induced HUS [9]. A further understanding of the mechanism(s) by which Stx crosses the intestinal epithelial barrier may lead to additional approaches to treat those infected with STEC, particularly since antibiotic therapy is contraindicated due to the increased risk for HUS [10]. Trans-cellular transport is the most common mechanism reported for Stx movement through the intestinal epithelium: purified Stxs move transcellularly across polarized colonic Gb3-negative T84 cells and Gb3-positive HCT-8 cells without disruption of the epithelial cell barrier [6,11,12,13,14,15]. In addition, infection of polarized T84 cells with an O157:H7 *E. coli* strain under microaerobic conditions was found to significantly increase the amount of Stx2a transported transcellularly [14]. Studies using intestinal epithelial cells also suggest that Stx1a can cross T84 cells after uptake by macropinocytosis [16,17]. This latter conclusion has been contested, however, as Tran et al. observed no evidence of toxin uptake by macropinocytosis [14]. Two distinct paracellular transport pathways have also been hypothesized for Stx translocation. The first method involves direct damage to the mucosa and epithelial barrier, as infection of polarized T84 cells with O157:H7 STEC strain CL56 (Stx1a+Stx2a+) caused increases in permeability for Stx, as noted by decreased transepithelial electrical resistance (TEER) values and disruption of zonula occludin (ZO-1) staining [18]. The second method entails paracellular trafficking of Stx by immune cells through the epithelial barrier as Stx has been shown to be taken up by polymorphonuclear leukocytes [19] and has been found in blood cell-derived microvesicles [20]. Collectively the literature suggests that the Stxs may employ different methods to traverse the intestinal epithelium. The contrasting results from the different studies points to the inherent variability of the experimental models that are used to study Stx translocation in vitro. Historically, most of the knowledge of Stx transport has been derived using monolayers of immortalized intestinal epithelial cells (e.g., Caco-2, HCT-8, T84). It has been difficult to detect Stx in mice after oral infection with STEC and oral gavage with Stxs requires 1000-fold greater levels of toxin than are required for lethal intoxication [21]. However, it is noteworthy that both Stx1a and Stx2a are disseminated from the gut of orally intoxicated mice and found in the kidneys [22].

The evolution of in vitro tissue models in recent years, however, has offered evidence that Stx behavior is modified in tissue models with greater physiological complexity than cultured monolayers. Recent work with human intestinal organoids (HIOs) composed of stem-cell-derived epithelial and mesenchymal cells demonstrated that exposure to purified Stx1a or Stx2a from either the luminal or medium side diminishes barrier function 48 h after Stx addition [23]. In contrast, enteroids grown as two-dimensional monolayers were only susceptible to Stx2a when overlaid on the apical side and barrier function was maintained for 15 days after Stx addition [23]. These results indicate that mesenchymal cells (e.g., myofibroblasts) in the HIOs play an important physiological role for the epithelial barrier. The same study demonstrated that both mesenchymal monolayers and enteroids were susceptible to cytotoxic damage from Stx through apoptotic and necrotic pathways, whereas HIOs demonstrated clusters of both necrosis and cell proliferation [23]. Similar physiological responses have also been demonstrated with STEC in HIOs, as a study comparing STEC versus commensal strains of *E. coli* found no barrier damage in the presence of the commensal strain [24] but showed a loss of epithelial structural integrity when exposed to an O157:H7 *E. coli* strain that produced Stx2a [24]. Such results highlight the need for advanced in vitro intestinal models to gain a more thorough understanding of Stx translocation mechanics, which will in turn advance studies of therapeutic strategies against Stx.

**Figure 1:**
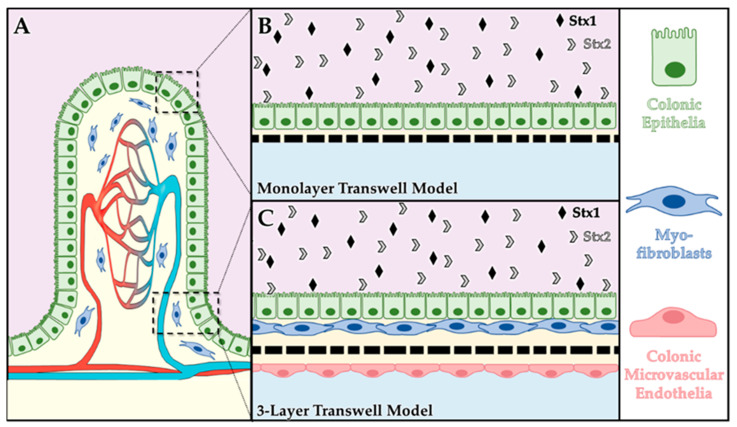
Conceptual illustrations of colonic transwell models. (**A**) Schematic depiction of a villus with key tissue layers of interest: the epithelial barrier (green), the extracellular matrix layer (ECM, yellow) with integrated myofibroblasts (blue), and the capillary bed (red to blue). The epithelial cell monolayer model (**B**) is composed of colonic epithelia (green) atop a deposited ECM (yellow) on the apical side of a tissue culture transwell. The three-layer model (**C**) is composed of colonic epithelia (green), myofibroblasts (blue), and ECM (yellow) on the apical side and ECM (yellow) and colonic microvascular endothelia (red) on the basolateral side of the transwell.

In this study, we assessed the capacity of purified Stx1a or Stx2a to translocate across intestinal epithelial cell barriers in two different in vitro tissue models (Figure 1): (i) a human primary colonic epithelial cell monolayer (Figure 1B) and (ii) a three-layer model consisting of human primary colonic epithelia, human mesenchymal-stem-cell (MSC)-derived myofibroblasts, and human primary colonic microvascular endothelia (Figure 1C). In model (i), the primary epithelial cell monolayer simulates the luminal layer of the intestine and provided cellular-level insights into Stx travel through and/or disruption of epithelial barriers. In model (ii), the three cellular layers were designed to simulate a simplified section of intestinal tissue, with an apical intestinal epithelial layer, a connective layer of myofibroblasts and extracellular matrix (ECM), and a basolateral vascular endothelial layer (Figure 1A). This model complements the former by providing insights into both cellular- and tissue-level interactions of Stx, including translocation through the ECM. We observed that purified Stx1a and Stx2a translocated across both model systems when applied to either the apical side or the basolateral side. Addition of an O26:H11 STEC strain that makes both Stx1a and Stx2a also resulted in modest toxin translocation. However, an O157:H7 isolate caused destruction of the model within 6 hours, so toxin translocation could not accurately be measured.

## 2. Results

In designing a cellular intestinal model to investigate Stx translocation, we selected three human-derived cell populations (i.e., colonic epithelia, myofibroblasts, and colonic microvascular endothelia) to represent three sections of tissue that play key roles in Stx translocation and tissue targeting (i.e., intestinal luminal lining, mesenchymal connective layer, vascular lining; see Figure 1 [4,5,25]). Prior to assembling the cells into multi-layer transwell cultures, our first steps were to ensure the cells could be co-cultured and could express appropriate genotypic and phenotypic markers in vitro.

### 2.1. Cell Model Validation

#### Quantitative Reverse Transcription PCR (RT-qPCR)

**Figure 2:**
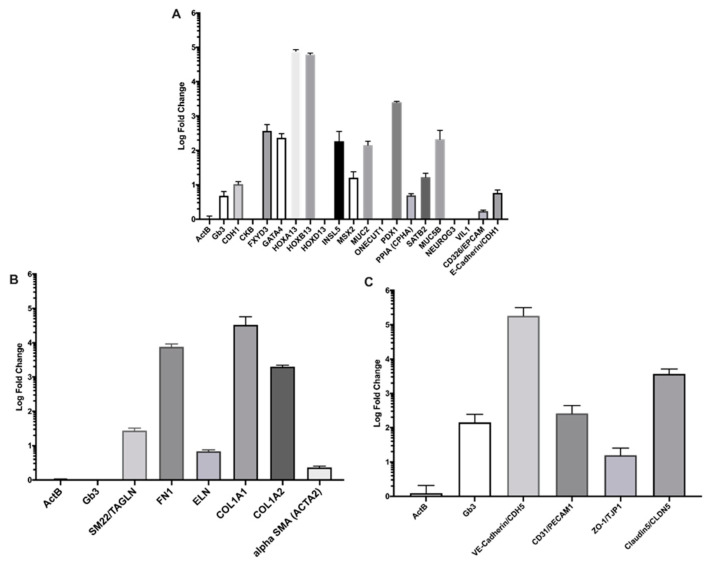
Log fold-change in gene expression relative to iPSCs. (**A**) Primary colonic epithelial cells, (**B**) MSC-differentiated myofibroblasts, and (**C**) primary colonic endothelial cells. ActB was used as a control. The full names of the referenced genes are defined in Supplementary Table S1.

As a preliminary performance metric for the in vitro cultured cells, we used RT-qPCR to compute the relative expression of several expected genetic markers for each cell line (see Supplementary Table S1 for gene names and primer sequences). Expression of each genetic sequence was computed relative to that of an undifferentiated induced pluripotent stem cell (iPSC) population. We used the beta (β)-actin (*ACTB*) gene as a control sequence that is conserved between all four cell populations. We also measured expression of Gb3 synthase which acts on lactosylceramide to generate Gb3, the Stx receptor. For each cell line, we found that nearly all the predicted markers were expressed at a higher rate than the reference population; see Figure 2. For the epithelial cell population, we found significant increases in gene expression for 15 of the 20 expected markers, and, notably, saw an over 0.5 log-fold increase in Gb3 synthase expression. This latter result agrees with previous findings of low levels of Gb3 on intestinal epithelial cells [6]. In the myofibroblast population, we saw increased expression of six expected myofibroblast markers which suggested proper differentiation, while the relative expression for Gb3 synthase was found to be negligible. Results for the endothelial cell population demonstrated an increased expression of all five genetic markers, with evidence of modest expression of Gb3 synthase at a 2 log-fold greater expression compared to the reference sample.

**Figure 3:**
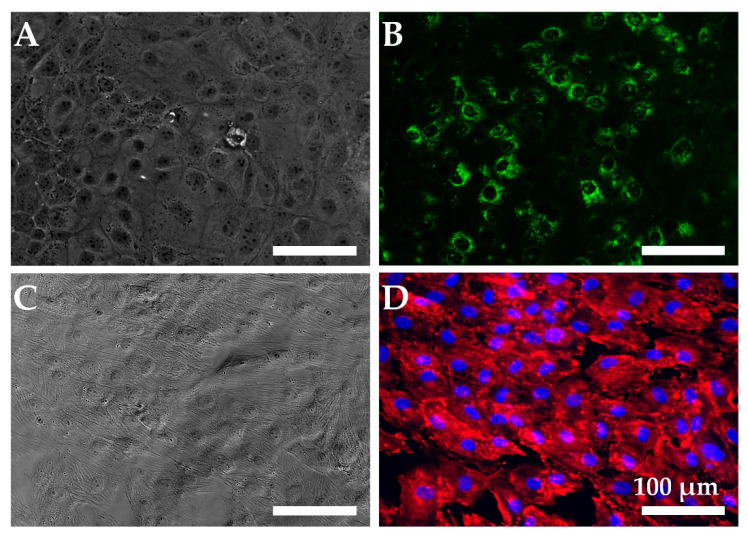
Cells used for the intestinal model express appropriate markers in monoculture. (**A**) Phase-contrast image and (**B**) fluorescence image of primary human colonic epithelial cells incubated with anti-Muc5B (green). (**C**) Phase-contrast and (**D**) fluorescence image of hMSC-derived myofibroblasts incubated with anti-αSMA (red) and counterstained with 4′,6-diamidino-2-phenylindole (DAPI, blue). Scale bars indicate 100 µm.

Beyond genotypic expression, we also leveraged immunostaining and histological staining to study the phenotypic expression of each of the cultured cell lines. Immunostaining of epithelial monolayers at confluence (Figure 3A,B) confirmed that mucin 5B (Muc5B), a mucin precursor common to intestinal epithelia, was expressed by the primary human colonic cells. A confluent monolayer of MSC-derived myofibroblasts (Figure 3C,D) demonstrated significant alpha-smooth muscle actin (α-SMA), expression, which is a marker of a mature myofibroblast [26]. As expected, we found that the myofibroblasts did not express Muc5B in monoculture, while the epithelial cells did exhibit some staining for α-SMA; see Supplemental Figure S1. Both cell types expressed ZO-1 in monoculture; see Supplemental Figure S1. We also used immunostaining to further evaluate the expression of Stx receptor Gb3 in colonic epithelial and endothelial cell monolayer cultures. Faint positive staining for Gb3 was observed for both cell types, consistent with the RT-qPCR results shown in Figure 2 and Supplemental Figure S2. 

**Figure 4:**
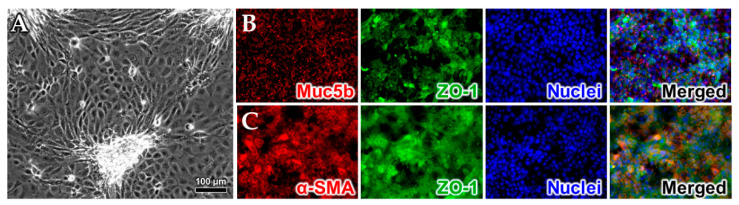
Co-cultured human colonic and human adipose-derived mesenchymal stem-cell (hAD-MSC)-derived myofibroblasts express appropriate markers. Phase-contrast image (**A**), and fluorescence images of cells incubated with antibodies specific for (**B**) Muc5b (red) and ZO-1 (green) or (**C**) αSMA (red) and ZO-1 (green). The cell nuclei were counterstained with DAPI (blue) and merged images of all three channels are displayed on the right.

**Figure 5:**
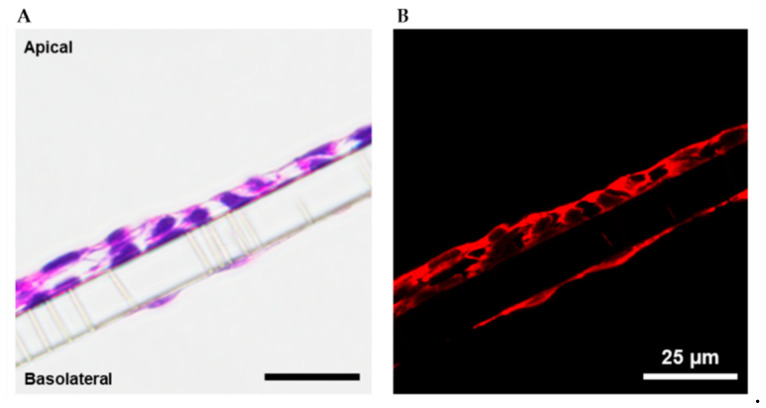
Cross section images of the three-layer intestinal model system. (**A**) H&E-stained layers depict the three layers of cells, while (**B**) fluorescence imaging of the stained cells offers greater resolution to visualize the endothelial layer.

To evaluate the capacity of the myofibroblast and epithelial cell lines to be co-cultured in direct contact, we seeded differentiated myofibroblasts onto a transwell membrane, cultured them to 80–90% confluence, and then seeded primary colonic epithelia directly over top. A 3:1 mixture of epithelial cell media and myofibroblast differentiation media was found to sustain cell viability during co-culture for a minimum of 2 weeks. Growth and expansion of the epithelial layer was tracked with phase-contrast microscopy. The microscopy indicated successful co-culture methods as the epithelial cells readily generated a cobblestone-like monolayer; see Figure 4A. Myofibroblasts were also distinguishable from epithelia in the first 3–5 days following seeding and were found to maintain a consistent stretched morphology as in monoculture. We consistently observed that the epithelial cells assembled into clustered structures upon confluence (Figure 4A) which may represent an early form of assembly into microvilli as seen with other colonoids [27,28], though we cannot rule out some cell overgrowth. To confirm that these two cell types retain their phenotypic expression when grown together, we performed immunostaining of the co-cultures for Muc5b, ZO-1, and α-SMA; see Figure 4B,C. The fluorescence images depicted in Figure 4B demonstrated the presence of Muc5b (red) and ZO-1 (green) with a blue 4′,6-diamidino-2-phenylindole (DAPI) counterstain of the nuclei. Similarly, the fluorescence images in Figure 4C demonstrated the presence of α-SMA (red) and ZO-1 (green) with a blue DAPI counterstain of the nuclei. Taken together these data suggest the presence of both cell types. Next, we expanded the co-culture to a three-layer model system in transwells, with myofibroblasts and epithelia on the apical side and primary colonic endothelia seeded on the basolateral side. Following a roughly two-week culture protocol, the three-layer models were fixed, paraffin embedded, mounted, and stained with hematoxylin and eosin (H&E) (Figure 5) to verify the presence of the three cultured layers. We evaluated the histological samples by brightfield and fluorescence imaging and observed a dual cell layer on the apical side of the transwell that represented the epithelial and myofibroblast layers. We also observed a thin layer that verified the presence of endothelial cells on the basolateral side of the membrane.

### 2.2. Exposure of Cell Models to Purified Stxs

Previous studies showed that Stxs are cytotoxic to intestinal epithelial cells such as HCT-8s at high doses [6]. Therefore, we determined the threshold for cytotoxicity of Stx1a and Stx2a on epithelial and endothelial monolayer cultures. Although we detected modest Gb3 levels on the primary epithelial cells by immunostaining and Gb3-synthase transcript by RT-qPCR, we found that the cells were not susceptible to a dosage more than 10^6^-times the Vero cell cytotoxic dose 50% (CD_50_) of Stx1a or Stx2a. The endothelial cells were also insensitive to Stx2a but, in contrast, were sensitive to Stx1a at high toxin concentrations. Approximately 200 ng Stx1a was required to observe one Vero cell CD_50_ on the endothelial cells, an amount that represents more than 10^5^ CD_50_s for Vero cells. Given the relative resistance of the cell lines in monoculture to the toxins, we proceeded to the translocation assays without concern for excessive cell death and barrier breakdown in the presence of purified Stx.

#### 2.2.1. Response of Primary Colonic Epithelial Monolayer to Purified Stx 

**Figure 6:**
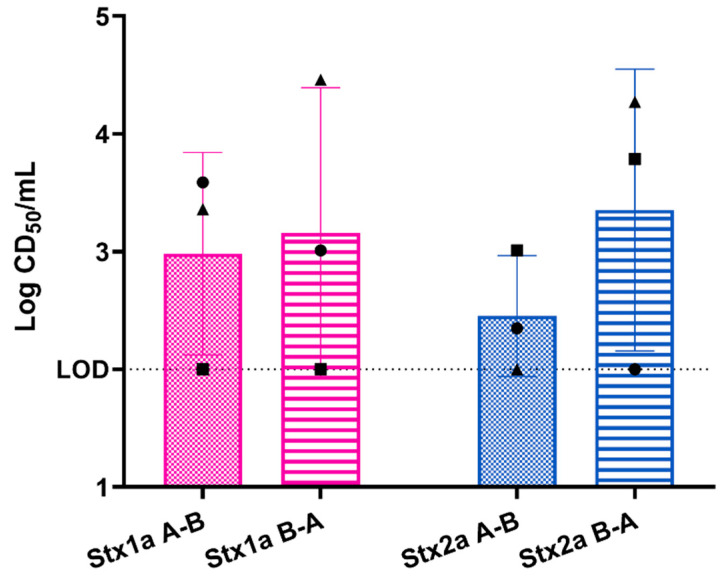
Stx1a and Stx2a translocated similarly across polarized primary epithelial cells when added to either the apical or basolateral side of the transwell. A-B: toxin was added to the apical side and measured from the basolateral side. B-A: toxin was added to the basolateral side and measured on the apical side. Each symbol represents the mean of two technical replicates. The experiment was performed three times. The bar is the overall mean. LOD = limit of detection. One-way analysis of variance (ANOVA) indicated no statistical differences among the values.

To understand Stx translocation through the intestinal epithelial barrier, we first leveraged a primary colonic epithelial cell monolayer as a comparator to published data with immortalized cell monolayers as well as data from our three-layer tissue model. When polarized primary epithelial cells were exposed to 400 ng purified Stx1a or Stx2a, both toxins translocated across the cells without significant change in the TEER. Unexpectedly, we observed that both Stx1a and Stx2a translocated across the monolayer regardless of the side (apical or basolateral) to which the toxin was applied, Figure 6. Similar levels of Stx1a and Stx2a as measured by CD_50_/mL translocated across the epithelial monolayers in either direction, which represented about 0.01% of the total toxin added to the model. The remainder of the toxin was detected in the compartment to which it was added, approximately 10^7^ CD_50_/mL. 

#### 2.2.2. Response of Three-Layer Colonic Co-Culture Model to Purified Stx

**Figure 7:**
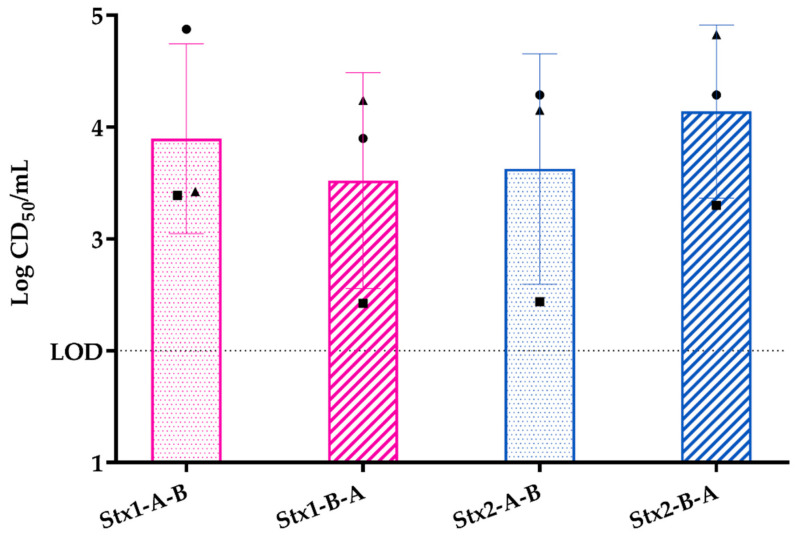
Translocation of purified Stx1a or Stx2a was similar across the three-layer tissue model as measured by total CD_50_ translocated. Each symbol represents the mean of two technical replicates. The experiment was repeated three times. The bar is the overall mean. One-way analysis of variance (ANOVA) indicated no statistical differences among the values.

As with the colonic epithelial monolayer, we exposed the three-layer tissue model to 400 ng purified Stx1a or Stx2a and found that both Stx1a and Stxa2 translocated across the model; see Figure 7. Over three biological replicates, a mean of 10^3.8^ CD_50_ of Stx1a translocated from the apical to the basolateral side of the model, a value that represents approximately 0.1 ng Stx1a or about 0.025% of the input. For Stx2a, the mean translocated was 10^3.6^ CD_50_ as measured on the Vero cells, a value that represents about 0.32 ng of Stx2a or about 0.09% of the input. Similar to the single-layer model, we measured toxin translocation in both directions, and found that almost all of the toxin stayed in the compartment to which it was added (10^7.4^ CD_50_/mL for Stx1a and 10^6.7^ CD_50_/mL for Stx2a on the apical side for this study). We were surprised that about a log more of Stx translocated across the three-layer model as crossed the single-layer model. This latter result suggests that the more complex model is more efficient at translocating toxin than the epithelial cell-only monolayer. 

### 2.3. Exposure of the Three-Layer Model to STEC

**Figure 8:**
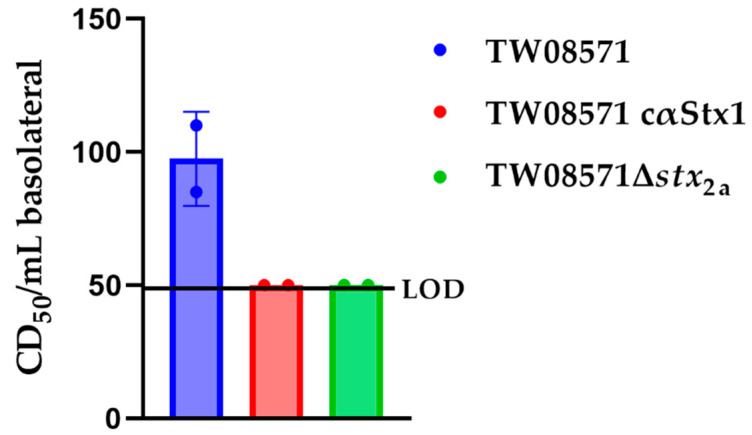
Toxin translocation across the three-layer model after 14 h of exposure to STEC strain TW08571, TW08671 with cαStx1, or an isogenic *stx*_2a_ deletion derivative of TW08571. The data points represent the mean of 2 technical replicates for each sample, *n* = 2 experiments.

To determine if we could measure toxin translocation in the presence of STEC, we exposed the three-layer model to O157:H7 STEC Stx2a+ strain 86-24 or O26:H11 Stx1a+ and Stx2a+ strain TW08571. At 6 h of exposure to the STEC, no toxin was detected in the basolateral compartment. We found over multiple studies that strain 86-24 and an intimin (encoded by *eae*) deletion derivative of 86-24 caused destruction of the model as evidenced by flow-through of fluorescein isothiocyanate (FITC)-dextran and recovery of bacteria in the basolateral compartment after 18 h of exposure to the STEC. Therefore, the rest of the bacterial exposure studies were performed with just strain TW08571. We incubated the model with TW08571, TW08571 plus anti-Stx1 antibody cαStx1, or TW08571Δ*stx*_2a_, for 14 h. In these preliminary studies, we observed low levels of Stx translocation across the three-layer model when TW08571 was added; see Figure 8. When we added either antibody to Stx1a along with TW08571 or used the *stx*_2a_ mutant strain, we observed no translocation of toxin. In one study not included in Figure 8, we observed an excess of FITC translocation, but we still found that toxin only translocated in the wells that had the wild-type strain, even though the toxin levels in the apical compartment were the same. 

## 3. Discussion

In this study we developed a three-layer tissue model that consists of colonic epithelial cell, myofibroblast, and colonic endothelial cell layers to study the translocation of Stxs. We found that Stx1a and Stx2a translocated similarly across polarized primary epithelial cells in terms of the number of CD_50_s that were detected on the opposite side of the transwell systems. However, because Stx2a is less toxic to Vero cells by 5–10 fold than Stx1a [29], these data suggest that overall, more Stx2a translocated in this model, a finding that contrasts with findings on polarized CaCo2A and HCT-8 cells in which 3- to 9-fold more Stx1a than Stx2a translocated [12]. Overall, we observed that approximately 0.01% of the toxin added to the epithelial cell model translocated, while about 0.09% of Stx2atranslocated in the three-layer model. Our results suggest that single-cell models may underestimate Stx translocation. The fact that we observed more Stx2a translocation than Stx1a is consistent with epidemiological data that Stx2a is more highly associated with the development of HUS than Stx1a [3]. Although it is unclear why only a small percentage of toxin translocates in the intestinal models, the data are consistent with the low level of HUS (which is the result of systemic intoxication) in people and consistent with the finding of toxin in the feces of animal models infected with STEC [30,31]. 

We observed over several studies that infection of the three-layer model with O157:H7 STEC strain 86-24 or 86-24Δ*eae* caused destruction of the model, but infection with O26:H11 strain TW08571 (*eae*+) did not. These results indicate that intimin (encoded by *eae*) is not necessary for model destruction. Taken together, our data also suggest that Stxs are not required for tissue destruction in the model since we did not observe destruction with purified toxins or with TW08571, which makes both Stx1a and Stx2a. It is possible that other genes such as *espB_*O157 and *etpD*, which are associated with more severe clinical outcome of STEC infection in children [32] and which are encoded by 86-24 but not TW08571, are required for the loss of model integrity. Consistent with our findings, Philpott et al. further found that infection of polarized T84 cells in Ussing chambers with an O157:H7 STEC altered barrier function in an Stx-independent manner after a 15 h incubation [13]. The authors of the latter study concluded that toxin was translocating by a transcellular route as toxin translocation was not found to increase even when barrier function was reduced. We similarly found that even when we observed a loss of barrier function in the three-layer model neither Stx1a nor Stx2a translocated as measured from TW08571⊗*stx*_2_ or TW08571 in the presence of cαStx1, respectively. We are not sure why Stx translocation from TW085781 seemed to require the presence of both toxins. We do not think that it is due to a higher level of toxin in the basolateral compartment because the overall toxin measured on the basolateral side was the same from TW08571 and TW08571⊗*stx*_2_ (not shown). It is also possible that translocation requires a certain level of toxin which was not reached under the conditions tested. 

We found that the Stxs translocated both the apical direction and the basolateral direction in our model, something also observed in other studies [11,12,23]. The meaning of the bi-directional movement is unclear but may simply indicate that the cellular mechanism of translocation is functional in either direction. We observed only low levels of the Stx receptor Gb3 on the colonic epithelial cells and endothelial cells, and both cell types were resistant to toxin action. These findings may indicate that the movement of toxin is independent of Gb3. 

Taken together, the three-layer system shows movement of functional toxin and could serve as a model system to test inhibitors of toxin trafficking from the intestine to system sites. In future studies, additional expansion of the tissue model could be performed to clarify the roles of specific physiological factors in the translocation of Stx. Such factors include (i) the microaerobic state of the intestinal lumen, which has been shown previously to modify cellular responses to Stx in vitro [14]; (ii) pulsatile flow, since the intestines undergo fluidic movement; or (iii) a system that integrates both microaerobic conditions and pulsatile flow in a single device. Finally, in future investigations, we could explore modifications to the methods from HIO studies [23,24] to generate a stem cell-derived enteroid layer with the requisite cell populations. Given the data generated in this study and the potential for enhancement of our in vitro intestinal model in the future, we anticipate this work will provide valuable contributions to advance the fields of Stx research, intestinal modeling, and in vitro modeling beyond the intestines.

## 4. Materials and Methods

### 4.1. Cell Culture

#### 4.1.1. Primary Cell Culture

Human primary colonic epithelial cells and human primary colonic microvascular endothelial cells were cultured following manufacturer’s instructions (Cell Biologics, Chicago, IL, USA). Cells were thawed and cultured in a T75 flask coated with gelatin-based coating solution (Cell Biologics) with either complete human epithelial cell media (Cell Biologics) or EGM-2 MV microvascular endothelial cell growth medium-2 BulletKit (Lonza, Walkersville, MD). Media was changed after initial plating, every 48 h when cells were <70% confluent and every 24 h when cells were >70% confluent. Cells were harvested at confluency for experimentation.

#### 4.1.2. Human Adipose-Derived Mesenchymal Stem/Stromal Cells

The hAD-MSC (RoosterBio, Inc. Frederick, MD, USA) were cultured following manufacturer’s instructions. Cells were thawed and cultured in a T225 flask in RoosterNourish-MSC medium. The medium was changed on day 4 at 70% confluency and the cells harvested on day 5 at >80% confluency.

#### 4.1.3. Myofibroblast Differentiation from hAD-MSCs

The myofibroblast differentiation protocol was derived from Desai et al. [26]. Briefly, 6-well tissue culture plates were coated with 10 µg/mL bovine collagen solution (Sigma-Aldrich, St. Louis, MO, USA) in 1X phosphate-buffered saline (PBS) for 1 h at 37 °C. After coating, the solution was removed and hAD-MSCs were plated at a density of 26,000 cells/cm^2^ in myofibroblast differentiation media consisting of DMEM:F12 (Invitrogen, Waltham, MA, USA), 0.1 mM MEM non-essential amino acids (ThermoFisher, Rockville, MD, USA), 1% penicillin/streptomycin (ThermoFisher), 1X GlutaMAX (ThermoFisher), 1X RPMI 1640 vitamins solution (Sigma), 1X ITS liquid media supplement (Sigma), 1 mM sodium pyruvate (Sigma), and 1 µg/mL glutathione (Sigma) for 1 h at 37 °C. After the cells were plated for 1 h, 1 ng/mL recombinant human TGF-β1 (R&D Systems, Minneapolis, MN, USA) was added to the media. Differentiation occurred over 4 days with media being changed (myofibroblast differentiation media + 1 ng/mL TGF-β1) after 48 h. Cells were then maintained in myofibroblast differentiation media without TGF-β1 while waiting for experimentation.

### 4.2. Transwell Model Systems

#### 4.2.1. Human Primary Colonic Epithelial Cell Monolayer Model

All epithelial model transwell experiments were conducted using 1.0 µm pore size polyethylene terephthalate (PET) membrane 12-well Millicell hanging cell culture inserts (MilliporeSigma, Rockville, MD, USA) in 12-well tissue culture plates. Culture inserts were coated with gelatin-based coating solution (Cell Biologics) for 10 min at 37 °C. After human primary colonic epithelial cells were grown to confluency and harvested for experimentation (as described in Section 4.1.1), coated inserts were seeded with 295,000 cells/cm^2^. Complete human epithelial cell media (Cell Biologics) was changed every 24 h until epithelial models were ready for experimentation on day 7. TEER measurements were collected using an EVOM2 Epithelial Volt/Ohm Meter (World Precision Instruments).

#### 4.2.2. Colonic 3-Layer Tissue Model

All tissue model transwell experiments were performed on 1.0 µm pore size PET membrane 12-well Millicell hanging cell culture inserts (MilliporeSigma) or on 1.0 µm pore size PET membrane 24-well Millicell hanging cell culture inserts (MilliporeSigma) in 12- or 24-well cell culture plates. Apical sides of culture inserts were coated with 10 µg/mL bovine collagen solution (Sigma) in gelatin-based coating solution (Cell Biologics) for 1 h at 37 °C and basal sides were coated with gelatin-based coating solution (Cell Biologics) for 10 min at 37 °C. The colonic tissue models were seeded in a step-wise fashion. First, the cell culture inserts were inverted and human primary colonic microvascular endothelial cells were seeded at a density of 194,000 cells/cm^2^ on the basal side. After incubation for 2 h at 37 °C, the inserts were placed right-side up and seeded with myofibroblasts at a density of 52,000 cells/cm^2^. Myofibroblast differentiation media without TGF-β1 as well as EGM-2 MV microvascular endothelial cell growth medium-2 Bullet Kit (Lonza) was changed every 24 h for 5 days. On day 5, human primary colonic epithelial cells were seeded at a density of 295,000 cells/cm^2^ on the cell culture insert apical side. A media change occurred every 24 h and consisted of a 3:1 mixture of complete human epithelial cell media (Cell Biologics) and myofibroblast differentiation media without TGF-β1 for the apical side and EGM-2 MV microvascular endothelial cell growth medium-2 BulletKit (Lonza) for the basal side. Experiments with tissue models were conducted 7–10 days after human primary colonic epithelial cell seeding.

### 4.3. Immunohistochemistry, Histology, and Microscopy

Tissues and cells were fixed in their specific culture environment for continuity. Single-layer monolayers were fixed and stained in 12-well cell culture plates and tissue models were fixed and stained on 1.0 µm pore size PET membrane transwell inserts in 12- or 24-well cell culture plates. Standard immunohistochemistry protocols were followed. Briefly, cells were rinsed in 1X PBS to remove culture media. They were then fixed with 4% paraformaldehyde in PBS for 10 min at room temperature before washing three times with PBS. For intracellular proteins, permeabilization was performed with 0.1% Triton X-100 in PBS for 10 min before washing samples in PBS three times at 5 min intervals. Non-specific binding of antibodies was blocked with a solution of 1% BSA, 22.52 mg/mL glycine, and 0.1% Tween 20 in PBS for 30 min. Samples were then incubated with the diluted primary antibody (Supplementary Table S2) in 1% BSA PBS overnight at 4 °C, then washed, and incubated with secondary antibody (Supplementary Table S2) in 1% BSA for 1 hour. If samples were stained using multiple primary and secondary antibody solutions it was performed in a sequential manner. Samples were then counterstained with DAPI (Invitrogen) for 1 min before washing and visualization. Samples in cell culture plates were imaged as such. Transwell samples were removed from the cell culture insert and placed on a standard glass slide and coverslip. All images were taken using a Nikon Eclipse Ts2 epifluorescence microscope at either 10× or 20×, then captured using NIS-Elements BR (Nikon, Minato City, Tokyo, Japan) software, and analyzed using ImageJ (NIH, Bethesda, MD, USA). Three-layer tissue models were prepared for histology using standard protocols. Cell culture media was aspirated from either side of the transwell and cells were rinsed with 1X PBS for 30 s. They were then fixed with 4% paraformaldehyde in PBS for 10 min at room temperature before washing three times with PBS. Following a dehydration step, fixed samples were embedded with paraffin wax and sectioned using a microtome. Sections were stained with hematoxylin and eosin and mounted on glass microscope slides with glass coverslips. Images of mounted samples were collected using a Nikon Eclipse Ts2 epifluorescent microscope at 40× magnification and analyzed using ImageJ.

### 4.4. RT-qPCR

RNA was extracted from the non-differentiated iPS cells, primary epithelial cells, hAD-MSC-derived myofibroblasts, and primary endothelial cells with the Zymo Research Quick-RNA Miniprep Kit following the manufacturer’s instructions. The RNA was converted to cDNA with the Qiagen QuantiTect Reverse Transcription Kit. Finally, reactions were set up in triplicate with the Qiagen QuantiTect SYBR green PCR kit. The RT-qPCR reactions were performed in a Rotor-Gene Q PCR Cycler (Qiagen, Hilden, Germany). Analysis of the relative fold gene expression of each sample was performed by calculating the ∆∆CT for each primer set with cDNA from the undifferentiated iPS cells as the control.

### 4.5. Stx Purification and Cytotoxicity Assay

The Stxs were purified by immuno-affinity as described previously [33]. The Vero cell cytotoxicity assay was performed as described previously [34]. Briefly, Vero cells were seeded into 96-well plates (10^5^ cells/mL), then overlaid with toxin 18–24 h later. After 48 h of incubation the plates were fixed in formalin, stained with crystal violet, washed with water and allowed to air dry. Absorbance readings were obtained at 590 nm. The CD_50_ for each sample was calculated as the inverse of the dilution that caused 50% cell killing relative to untreated cells.

### 4.6. Exposure of Transwells to Purified Stxs or to STEC

Purified Stx1 or Stx2 were diluted in appropriate media to a final concentration of 500 ng in 800 µL to add to the transwells. Fresh media was added to the apical and basolateral sides of the transwells and TEER measurements were taken and recorded. The fresh media was removed and the toxin samples were added in duplicate. Transwells were incubated at 37 °C, 5% CO_2_ for 5 h, then a final TEER measurement was taken and recorded. Samples were removed and stored at −20°C until the Vero cell cytotoxicity assay could be performed. 

STEC strains used for this study are listed in Table 1. The bacteria were grown in Luria broth overnight and approximately 10^3^ CFU were added to the transwells. After 6, 14, or 18 h of incubation, samples were taken from the apical and basolateral compartments for CFU enumeration and measurement of Stx levels. STEC strains 86-24, TW08571, TW08571Δ*stx*_2a_ were used for these studies. STEC strain TW08571Δ*stx*_2a_ was generated by amplifying an stx_2a_ gene with an internal deletion and an inserted chloramphenicol resistance cassette from strain JH2010 [31] with primers 2aSacIF (ATGCGAGCTCACTCATAATCGCCAGGTCGC) and 2aSacIR (ATGCGAGCTCCCCTGCTATGAGAGGCCTTG). After digestion with SacI, the PCR product was ligated to similarly digested suicide vector pCVD442 and the mutant strain generated by the method described [35]. We were unable to generate an stx_1a_ mutant derivative of TW08571 despite numerous attempts. An antibody to Stx1, cαStx1 [36], was used in some studies at a concentration of 0.5 mg/mL. 

For colonic tissue models, FITC-dextran with an average molecular weight of 3000–5000 was used to test permeability in addition to TEER measurements. FITC-dextran was diluted to 78 µg/mL in 800 µL of the Stx2 toxin sample that was applied to the transwells. When the assay was complete, 100 µL from each sample (both apical and basolateral sides) were added to a Corning 96-well solid black U-bottomed plate. Samples were read using a GloMax-Multi+ Detection System (Promega Corp., Madison, WI, USA) with a Blue optical kit (Ex: 490 nm, Em: 510–570 nm).

### 4.7. Statistical Analyses

All statistical analysis was performed with GraphPadPrism v9.4.1 for Windows (GraphPad Software, LLC, La Jolla, CA, USA). 

## Figures and Tables

**Table 1:** STEC strains used in this study.

Strain	Serotype, Toxin Type(s), *eae* Presence	Reference
86-24	O157:H7, Stx2a, *eae*+	[37]
86-24*eae*Δ10	O157:H7, Stx2a, *eae*-	[38]
TW08571	O26:H11, Stx1a + Stx2a, *eae*+	[34]
TW08571Δ*stx*_2a_	O26:H11, Stx1a; *eae*+	This study

## Data Availability

All data are contained within the article.

## Supplementary Materials

The following supporting information can be downloaded at: https://www.mdpi.com/article/10.3390/toxins15030207/s1, Figure S1: Immunofluorescence images showing Muc5B, αSMA, and ZO-1 staining in colonic epithelial cells and hMSC-derived myofibroblasts. Figure S2: Immunofluorescence images showing Gb3 expression in colonic epithelial cells and microvascular endothelial cells. Table S1: Gene names and primer sequences for all genes evaluated with RT-qPCR; Table S2: Immunofluorescence antibody sources and dilutions.

## References

[1] Bruyand M., Mariani-Kurkdjian P., Gouali M., de Valk H., King L.A., Le Hello S., Bonacorsi S., Loirat C. (2018). Hemolytic uremic syndrome due to Shiga toxin-producing *Escherichia coli* infection. Med. Mal. Infect..

[2] Biernbaum E.N., Kudva I.T. (2022). AB(5) Enterotoxin-Mediated Pathogenesis: Perspectives Gleaned from Shiga Toxins. Toxins.

[3] Scheutz F., Teel L.D., Beutin L., Pierard D., Buvens G., Karch H., Mellmann A., Caprioli A., Tozzoli R., Morabito S. (2012). Multicenter evaluation of a sequence-based protocol for subtyping Shiga toxins and standardizing Stx nomenclature. J. Clin. Microbiol..

[4] Melton-Celsa A.R. (2014). Shiga toxin (Stx) classification, structure, and function. Microbiol. Spectr..

[5] Detzner J., Pohlentz G., Müthing J. (2022). Enterohemorrhagic *Escherichia coli* and a Fresh View on Shiga Toxin-Binding Glycosphingolipids of Primary Human Kidney and Colon Epithelial Cells and Their Toxin Susceptibility. Int. J. Mol. Sci..

[6] Zumbrun S.D., Hanson L., Sinclair J.F., Freedy J., Melton-Celsa A.R., Rodriguez-Canales J., Hanson J.C., O’Brien A.D. (2010). Human intestinal tissue and cultured colonic cells contain globotriaosylceramide synthase mRNA and the alternate Shiga toxin receptor globotetraosylceramide. Infect. Immun..

[7] Steil D., Schepers C.L., Pohlentz G., Legros N., Runde J., Humpf H.U., Karch H., Müthing J. (2015). Shiga toxin glycosphingolipid receptors of Vero-B4 kidney epithelial cells and their membrane microdomain lipid environment. J. Lipid Res..

[8] Brigotti M., Carnicelli D., Arfilli V., Tamassia N., Borsetti F., Fabbri E., Tazzari P.L., Ricci F., Pagliaro P., Spisni E. (2013). Identification of TLR4 as the receptor that recognizes Shiga toxins in human neutrophils. J. Immunol..

[9] Mühlen S., Dersch P. (2020). Treatment Strategies for Infections With Shiga Toxin-Producing *Escherichia coli*. Front. Cell. Infect. Microbiol..

[10] Tarr P.I., Freedman S.B. (2022). Why antibiotics should not be used to treat Shiga toxin-producing *Escherichia coli* infections. Curr. Opin. Gastroenterol..

[11] Acheson D.W., Moore R., De Breucker S., Lincicome L., Jacewicz M., Skutelsky E., Keusch G.T. (1996). Translocation of Shiga toxin across polarized intestinal cells in tissue culture. Infect. Immun..

[12] Hurley B.P., Jacewicz M., Thorpe C.M., Lincicome L.L., King A.J., Keusch G.T., Acheson D.W. (1999). Shiga toxins 1 and 2 translocate differently across polarized intestinal epithelial cells. Infect. Immun..

[13] Philpott D.J., Ackerley C.A., Kiliaan A.J., Karmali M.A., Perdue M.H., Sherman P.M. (1997). Translocation of verotoxin-1 across T84 monolayers: Mechanism of bacterial toxin penetration of epithelium. Am. J. Physiol..

[14] Tran S.L., Billoud L., Lewis S.B., Phillips A.D., Schüller S. (2014). Shiga toxin production and translocation during microaerobic human colonic infection with Shiga toxin-producing *E. coli* O157:H7 and O104:H4. Cell. Microbiol..

[15] Russo L.M., Melton-Celsa A.R., Smith M.J., O’Brien A.D. (2014). Comparisons of native Shiga toxins (Stxs) type 1 and 2 with chimeric toxins indicate that the source of the binding subunit dictates degree of toxicity. PLoS ONE.

[16] Lukyanenko V., Malyukova I., Hubbard A., Delannoy M., Boedeker E., Zhu C., Cebotaru L., Kovbasnjuk O. (2011). Enterohemorrhagic *Escherichia coli* infection stimulates Shiga toxin 1 macropinocytosis and transcytosis across intestinal epithelial cells. Am. J. Physiol. Cell Physiol..

[17] Malyukova I., Murray K.F., Zhu C., Boedeker E., Kane A., Patterson K., Peterson J.R., Donowitz M., Kovbasnjuk O. (2009). Macropinocytosis in Shiga toxin 1 uptake by human intestinal epithelial cells and transcellular transcytosis. Am. J. Physiol. Gastrointest. Liver Physiol..

[18] Philpott D.J., McKay D.M., Mak W., Perdue M.H., Sherman P.M. (1998). Signal transduction pathways involved in enterohemorrhagic *Escherichia coli*-induced alterations in T84 epithelial permeability. Infect. Immun..

[19] Te Loo D.M., Monnens L.A., van Der Velden T.J., Vermeer M.A., Preyers F., Demacker P.N., van Den Heuvel L.P., van Hinsbergh V.W. (2000). Binding and transfer of verocytotoxin by polymorphonuclear leukocytes in hemolytic uremic syndrome. Blood.

[20] Ståhl A.L., Arvidsson I., Johansson K.E., Chromek M., Rebetz J., Loos S., Kristoffersson A.C., Békássy Z.D., Mörgelin M., Karpman D. (2015). A novel mechanism of bacterial toxin transfer within host blood cell-derived microvesicles. PLoS Pathog..

[21] Russo L.M., Melton-Celsa A.R., Smith M.A., Smith M.J., O’Brien A.D. (2014). Oral intoxication of mice with Shiga toxin type 2a (Stx2a) and protection by anti-Stx2a monoclonal antibody 11E10. Infect. Immun..

[22] Russo L.M., Melton-Celsa A.R., O’Brien A.D. (2016). Shiga Toxin (Stx) Type 1a Reduces the Oral Toxicity of Stx Type 2a. J. Infect. Dis..

[23] Pradhan S., Karve S.S., Weiss A.A., Hawkins J., Poling H.M., Helmrath M.A., Wells J.M., McCauley H.A. (2020). Tissue Responses to Shiga Toxin in Human Intestinal Organoids. Cell. Mol. Gastroenterol. Hepatol..

[24] Karve S.S., Pradhan S., Ward D.V., Weiss A.A. (2017). Intestinal organoids model human responses to infection by commensal and Shiga toxin producing *Escherichia coli*. PLoS ONE.

[25] Ray P., Acheson D., Chitrakar R., Cnaan A., Gibbs K., Hirschman G.H., Christen E., Trachtman H. (2002). Basic fibroblast growth factor among children with diarrhea-associated hemolytic uremic syndrome. J. Am. Soc. Nephrol..

[26] Desai V.D., Hsia H.C., Schwarzbauer J.E. (2014). Reversible modulation of myofibroblast differentiation in adipose-derived mesenchymal stem cells. PLoS ONE.

[27] Forbester J.L., Goulding D., Vallier L., Hannan N., Hale C., Pickard D., Mukhopadhyay S., Dougan G. (2015). Interaction of *Salmonella enterica* Serovar Typhimurium with Intestinal Organoids Derived from Human Induced Pluripotent Stem Cells. Infect. Immun..

[28] In J., Foulke-Abel J., Zachos N.C., Hansen A.M., Kaper J.B., Bernstein H.D., Halushka M., Blutt S., Estes M.K., Donowitz M. (2016). Enterohemorrhagic *Escherichia coli* reduce mucus and intermicrovillar bridges in human stem cell-derived colonoids. Cell. Mol. Gastroenterol. Hepatol..

[29] Tesh V.L., Burris J.A., Owens J.W., Gordon V.M., Wadolkowski E.A., O’Brien A.D., Samuel J.E. (1993). Comparison of the relative toxicities of Shiga-like toxins type I and type II for mice. Infect. Immun..

[30] Zhang Q., Donohue-Rolfe A., Krautz-Peterson G., Sevo M., Parry N., Abeijon C., Tzipori S. (2009). Gnotobiotic piglet infection model for evaluating the safe use of antibiotics against *Escherichia coli* O157:H7 infection. J. Infect. Dis..

[31] Hauser J.R., Atitkar R.R., Petro C.D., Lindsey R.L., Strockbine N., O’Brien A.D., Melton-Celsa A.R. (2020). The Virulence of *Escherichia coli* O157:H7 Isolates in Mice Depends on Shiga Toxin Type 2a (Stx2a)-Induction and High Levels of Stx2a in Stool. Front. Cell. Infect. Microbiol..

[32] Matussek A., Jernberg C., Einemo I.M., Monecke S., Ehricht R., Engelmann I., Löfgren S., Mernelius S. (2017). Genetic makeup of Shiga toxin-producing *Escherichia coli* in relation to clinical symptoms and duration of shedding: A microarray analysis of isolates from Swedish children. Eur. J. Clin. Microbiol. Infect. Dis..

[33] Melton-Celsa A.R., O’Brien A.D., Aktories K., Just I. (2000). Shiga toxins of *Shigella dysenteriae* and *Escherichia coli*. Handbook of Experimental Pharmacology.

[34] Petro C.D., Trojnar E., Sinclair J., Liu Z.M., Smith M., O’Brien A.D., Melton-Celsa A. (2019). Shiga toxin (Stx) type 1a reduces the toxicity of the more potent Stx2a in vivo and in vitro. Infect. Immun..

[35] Donnenberg M.S., Kaper J.B. (1991). Construction of an *eae* deletion mutant of enteropathogenic *Escherichia coli* by using a positive-selection suicide vector. Infect. Immun..

[36] Edwards A.C., Melton-Celsa A.R., Arbuthnott K., Stinson J.R., Schmitt C.K., Wong H.C., O’Brien A.D., Kaper J.B., O’Brien A.D. (1998). Vero cell neutralization and mouse protective efficacy of humanized monoclonal antibodies against *Escherichia coli* toxins Stx1 and Stx2. Escherichia coli O157:H7 and Other Shiga Toxin-Producing E. coli Strains.

[37] Ostroff S.M., Griffin P.M., Tauxe R.V., Shipman L.D., Greene K.D., Wells J.G., Lewis J.H., Blake P.A., Kobayashi J.M. (1990). A statewide outbreak of *Escherichia coli* O157:H7 infections in Washington State. Am. J. Epidemiol..

[38] McKee M.L., Melton-Celsa A.R., Moxley R.A., Francis D.H., O’Brien A.D. (1995). Enterohemorrhagic *Escherichia coli* O157:H7 requires intimin to colonize the gnotobiotic pig intestine and to adhere to HEp-2 cells. Infect. Immun..

