# Supplementary material for: Shiga Toxin (Stx) Type 1a and Stx2a Translocate through a Three-Layer Intestinal Model
Source: Toxins (Basel). 2023 Mar 9;15(3):207. doi: 10.3390/toxins15030207 (PMC10054274; doi:10.3390/toxins15030207)
Supplement: Supplementary file 1 [file toxins-15-00207-s001.zip › toxins-2207168-supplementary.pdf]

Supplementary Information

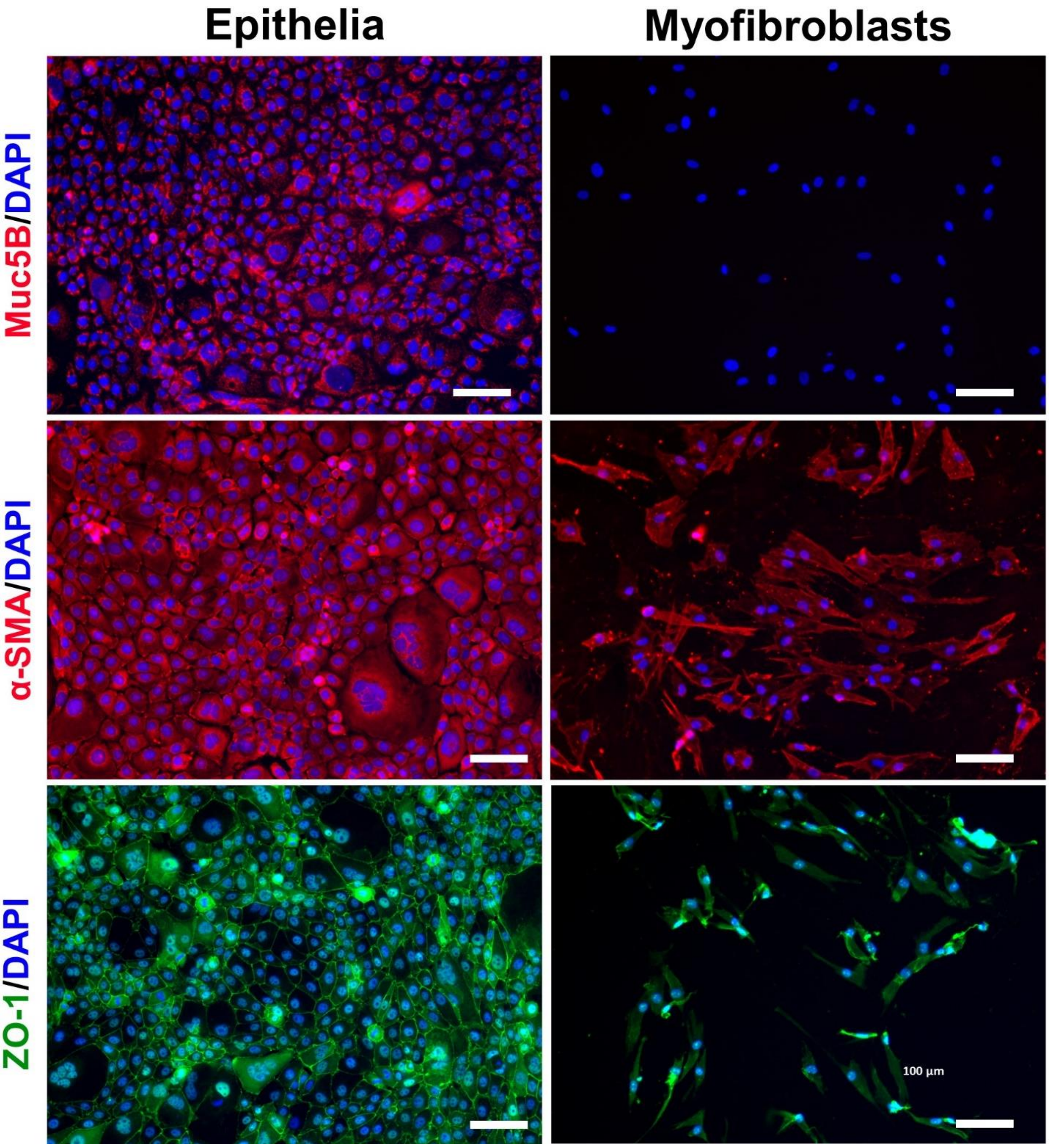

Supplemental Figure S1. Immunofluorescence images showing Muc5B expression (top row), αSMA (middle row) or ZO-1 (bottom row) in monolayers of primary colonic epithelial cells and hMSC-derived myofibroblasts. The cell nuclei were counterstained with DAPI as a visual aid.

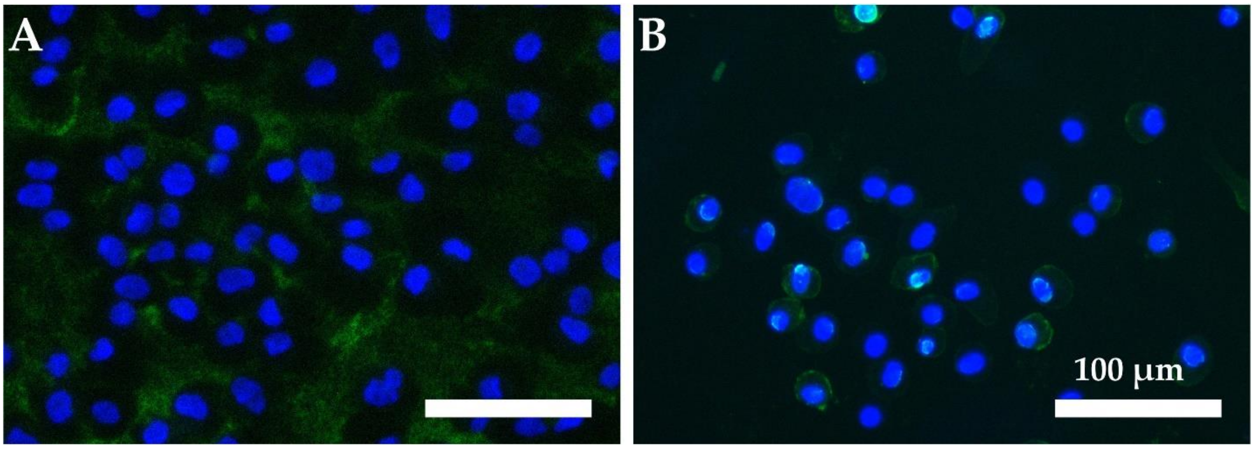

Supplemental Figure S2. Immunofluorescence images showing Gb3 expression (green) in monolayers of (A) primary colonic epithelial cells and (B) primary colonic microvascular endothelial cells. The cell nuclei were counterstained with DAPI as a visual aid.

Supplementary Table S1: Gene names and primer sequences for all genes evaluated with RT-qPCR

Epithelial Genes			
**Gene**	**Official Name**	**Forward Primer Sequence (5'--3')**	**Reverse Primer Sequence (5'--3')**
ACTB	Beta Actin	CACCATTGGCAATGAGCGGTTC	AGGTCTTTGCGGATGTCCACGT
GB3	Globotriaosylceramide	ACTTCATTGTTGATCTTGCATG	CGACGAATTCCCAGCTAAAC
CDH1	Cadherin 1	GACCGGTGCAATCTTCAAA	TTGACGCCGAGAGCTACAC
CKB	Creatine Kinase B	CCCACACCAGGAAGGTCTTA	CCTCTTCGACAAGCCCGT
FXYD3	FXYD domain containing ion transport regulator 3	AGGGTCACCTTCTGCATGTC	CTTCGGATAAACGCAGGACT
GATA4	GATA binding protein 4	TAGCCCCACAGTTGACACAC	GTCCTGCACAGCCTGCC
HOXA13	Homeobox A 13	GCACCTTGGTATAAGGCACG	CCTCTGGAAGTCCACTCTGC
HOXB13	Homeobox B 13	GCTGTACGGAATGCGTTTCT	AACCCACCAGGTCCCTTTT
HOXD13	Homeobox D 13	CCTCTTCGGTAGACGCACAT	CAGGTGTACTGCACCAAGGA
INSL5	Insulin like 5	GAAGGTTTTGCGCTGGATT	GATCCCTCAAGCTCAGCAAG
MSX2	Msh Homeobox 2	GGTCTTGTGTTTCCTCAGGG	AAATTCAGAAGATGGAGCGG
MUC2	Mucin 2	TGTAGGCATCGCTCTTCTCA	GACACCATCTACCTCACCCG
ONECUT1	One Cut Homeobox 1	TTTTTGGGTGTGTTGCCTCT	AGACCTTCCGGAGGATGTG
PDX1	Pancreatic and Duodenal Homeobox 1	CGTCCGCTTGTTCTCCTC	CCTTTCCCATGGATGAAGTC
PPIA (CPHA)	Peptidylprolyl Isomerase A	CCCACCGTGTTCTTCGACATT	GGACCCGTATGCTTTAGGATGA
SATB2	SATB Homeobox 2	CCACCTTCCCAGCTTGATT	TTAGCCAGCTGGTGGAGACT
MUC5B	Mucin 5b	GCCTACGAGGACTTCAACGTC	CCTTGATGACAACACGGGTGA
NEUROG3	Neurogenin 3	CTAAGAGCGAGTTGGCACTGA	GAGGTTGTGCATTCGATTGCG
VIL1	Villin 1	CTGAGCGCCCAAGTCAAAG	AGCAGTCACCATCGAAGAAGC
CD326/EPCAM	Epithelial Cell Adhesion Molecule	TGATCCTGACTGCGATGAGAG	CTTGTCTGTTCTTCTGACCCC

E-Cadherin/CDH1	Cadherin 1, E-cadherin	CGAGAGCTACACGTTCACGG	GGGTGTCGAGGGAAAAATAGG
**Myofibroblast Genes**			
**Gene**	**Official Name**	**Forward Primer Sequence (5'--3')**	**Reverse Primer Sequence (5'--3')**
ACTB	Beta Actin	CACCATTGGCAATGAGCGGTTC	AGGTCTTTGCGGATGTCCACGT
GB3	Globotriaosylceramide	ACTTCATTGTTGATCTTGCATG	CGACGAATTCCCAGCTAAAC
SM22/TAGLN	Spicule Matrix Protein 22, Transgelin	CCGTGGAGATCCCAACTGG	CCATCTGAAGGCCAATGACAT
FN1	Fibronectin 1	CGGTGGCTGTCAGTCAAAG	AAACCTCGGCTTCCTCCATAA
ELN	Elastin	GCAGGAGTTAAGCCCAAGG	TGTAGGGCAGTCCATAGCCA
COL1A1	Collagen Type 1, α1 chain	GAGGGCCAAGACGAAGACATC	CAGATCACGTCATCGCACAAC
COL1A2	Collagen Type 1, α2 chain	GAGCGGTAACAAGGGTGAGC	CTTCCCCATTAGGGCCTCTC
alpha SMA (ACTA2)	Actin α2, smooth muscle	AAAAGACAGCTACGTGGTGA	GCCATGTTCTATCGGGTACTTC
**Endothelial Genes**			
**Gene**	**Official Name**	**Primer Sequence (5'--3')**	**Reverse Primer Sequence (5'--3')**
ACTB	Beta Actin	CACCATTGGCAATGAGCGGTTC	AGGTCTTTGCGGATGTCCACGT
GB3	Globotriaosylceramide	ACTTCATTGTTGATCTTGCATG	CGACGAATTCCCAGCTAAAC
VE-Cadherin/CDH5	Cadherin 5, VE-Cadherin	TTGGAACCAGATGCACATTGAT	TCTTGCGACTCACGCTTGAC
CD31/PECAM1	Platelet and Endothelial Cell Adhesion Molecule	AACAGTGTTGACATGAAGAGCC	TGTAAAACAGCACGTCATCCTT
ZO-1/TJP1	Zonula Occludin 1	CAACATACAGTGACGCTTCACA	CACTATTGACGTTTCCCCACTC
Claudin 5/CLDN5	Claudin 5	CTCTGCTGGTTCGCCAACAT	CAGCTCGTACTTCTGCGACA

Supplementary Table S2: Immunofluorescence antibody sources and dilutions

**Primary Antibody**	**Source**	**Dilution**
Mucin 5b -- Mouse	Santa Cruz	1:100
Anti-α-SMA -- Mouse	Sigma	1:200
Anti-ZO1 -- Rabbit	Invitrogen	1:50
DAPI	Invitrogen	300 nM
Anti-CD77 -- Mouse	BD Biosciences	1:1000
**Secondary Antibody**	**Source**	**Dilution**
Goat anti-mouse Alexa Fluor 488	Invitrogen	1:500
Goat anti-rabbit Alexa Fluor 488	Invitrogen	1:500
Donkey anti-rabbit Alexa Fluor 555	Invitrogen	1:500

Donkey anti-mouse Alexa Fluor 555	Invitrogen	1:500
